# Internal cross-linked polymeric nanoparticles with dual sensitivity for combination therapy of muscle-invasive bladder cancer

**DOI:** 10.1186/s12951-020-00686-3

**Published:** 2020-09-04

**Authors:** Guanchen Zhu, Kaikai Wang, Haixiang Qin, Xiaozhi Zhao, Wei Chen, Linfeng Xu, Wenmin Cao, Hongqian Guo

**Affiliations:** 1 Department of Urology, Affiliated Drum Tower Hospital, Medical School of Nanjing University, Nanjing, 210009 China; 2 School of Pharmacy, Nantong University, Nantong, 226001 China

**Keywords:** Dual-sensitivity, Nanoparticle delivery, Bladder cancer, Chemotherapy, Photothermal therapy

## Abstract

**Background:**

Chemotherapy is a standard cancer treatment which uses anti-cancer drugs to destroy or slow the growth of cancer cells. However, chemotherapy has limited therapeutic effects in bladder cancer. One of the reasons of this resistance to chemotherapy is that higher levels of glutathione in invasive bladder cancer cells. We have fabricated nanoparticles that respond to high concentrations of glutathione and near-infrared laser irradiation in order to increase the drug accumulation at the tumor sites and combine chemotherapy with photothermal therapy to overcome the challenges of bladder cancer treatment.

**Methods:**

The DOX&IR780@PEG-PCL-SS NPs were prepared by co-precipitation method. We investigated the tumor targeting capability of NPs in vitro and in vivo. The orthotopic bladder cancer model in C57BL/6 mice was established for in vivo study and the photothermal effects and therapeutic efficacy of NPs were evaluated.

**Results:**

The DOX&IR780@PEG-PCL-SS NPs were synthesized using internal cross-linking strategy to increase the stability of nanoparticles. Nanoparticles can be ingested by tumor cells in a short time. The DOX&IR780@PEG-PCL-SS NPs have dual sensitivity to high levels of glutathione in bladder cancer cells and near-infrared laser irradiation. Glutathione triggers chemical structural changes of nanoparticles and preliminarily releases drugs, Near-infrared laser irradiation can promote the complete release of the drugs from the nanoparticles and induce a photothermal effect, leading to destroying the tumor cells. Given the excellent tumor-targeting ability and negligible toxicity to normal tissue, DOX&IR780@PEG-PCL-SS NPs can greatly increase the concentration of the anti-cancer drugs in tumor cells. The mice treated with DOX&IR780@PEG-PCL-SS NPs have a significant reduction in tumor volume. The DOX&IR780@PEG-PCL-SS NPs can be tracked by in vivo imaging system and have good tumor targeting ability, to facilitate our assessment during the experiment.

**Conclusion:**

A nanoparticle delivery system with dual sensitivity to glutathione and near-infrared laser irradiation was developed for delivering IR780 and DOX. Chemo-photothermal synergistic therapy of both primary bladder cancer and their metastases was achieved using this advanced delivery system.
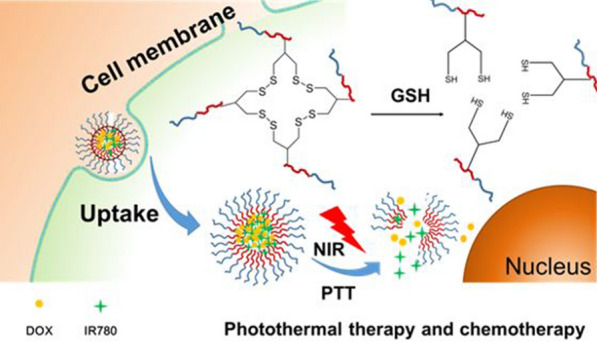

## Introduction

Bladder cancer, usually originates from the epithelial lining of a urinary bladder, is one of the most common malignancies of the urinary system [1–3]. Approximately 75% of patients present non-muscle invasive bladder cancer (NMIBC) and 20% present muscle invasive bladder cancer (MIBC). NMIBC recurs at a rate of 50–80% and has a 14% chance of progression to muscle-invasive cancer after transurethral resection (TUR) alone [4]. Radical total cystectomy is almost inevitable in muscle-invasive bladder cancer. However, patients with bladder tumor resection still have a high risk of death and will reduce the quality of life of patients [5]. Therefore, radical surgery combined with systemic chemotherapy is routinely used for muscle-invasive bladder cancer [6]. Photothermal therapy (PTT) shows strong promise for treating tumors. PTT makes photosensitizers generate heat from light absorption, which can cause cellular necrosis and apoptosis and shows a high therapeutic efficacy for tumor ablation [7–10]. However, current photosensitizers have limitations including poor selectivity, poor bioavailability and low biocompatibility, which hinders its clinical use [11, 12]. Therefore, it is of great significance to overcome these limitations and improve the treatment effect of bladder cancer.

According to previous studies, bladder cancer tissue has a high level of reduced glutathione (GSH) in the tumor microenvironment [13, 14]. GSH can neutralize oxidative stress and repair DNA damage caused by chemotherapy. In addition, GSH is involved in the excretion of chemotherapy drugs from tumor cells, resulting in a decrease in drug accumulation in tumor cells. It is also worth highlighting the correlations between GSH levels and aggressiveness of bladder cancer cells [15]. Therefore, GSH plays an important role in the resistance to chemotherapy of bladder cancer.

**Fig. 1:**
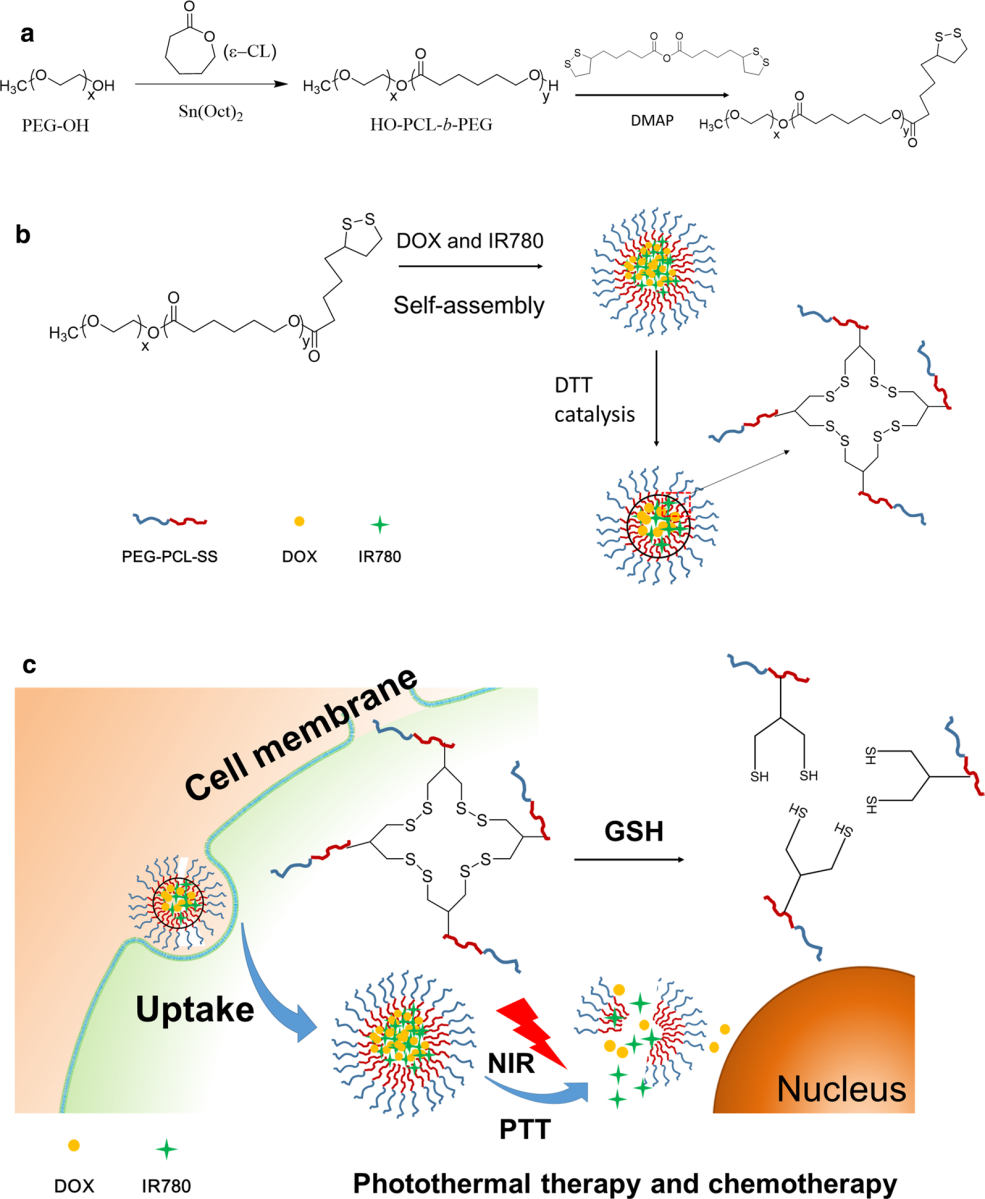
**a** Synthetic route of PEG-PCL-SS polymer. **b** Schematic illustration of the preparation of IR780 and DOX-loaded PEG-PCL-SS nanoparticles. **c** Chemo-photothermal therapy of cross-linked PEG-PCL-SS nanoparticles with reductive sensitivity and NIR laser-controlled drug release

To overcome the resistance of the treatment, photothermal therapy and chemotherapy were combined in the comprehensive treatment of bladder cancer to improve the therapeutic effects [16, 17]. In this study, we use doxorubicin (DOX) and IR780 (a near infrared dye) to form micelles self-assembled with PEG-PCL-SS by hydrophobic interaction, and disulfide bond rearrangement under disulfide (DTT) catalysis conditions, further internal cross-linking, forming the final nanoparticle. This nanoparticle system has the characteristics of long circulation and low toxicity, and is easily ingested by tumor cells. After the nanoparticles are taken up into the cells, the disulfide bonds in the nanoparticles are reduced to sulfhydryl groups due to the high GSH concentration in the cells. The hydrophobic interaction force of the nanoparticles is then weakened, in which triggers the initial disintegration of the nanoparticles (Fig. 1c). In addition, IR780 can generate heat under the excitation of NIR laser to further induce release the drugs [18]. Thereby the nanoparticle delivery system achieves the combined therapeutic effects of PTT and chemotherapy in order to overcome the practical problems encountered clinically and improve the treatment of bladder cancer.

## Materials and methods

### Chemicals and materials

Doxorubicin and ε-Caprolactone were purchased from Aladdin co. (Shanghai, China). IR780 was purchased from Sigma-Aldrich co. (St. Louis, USA), PEG was obtained from Peng Sheng Biological Co., Ltd (Shanghai, China). Poly(lactic-*co*-glycolic acid) (PLGA) and human serum albumin was obtained from Aladdin co. (Shanghai, China). MTT Cell Proliferation and Cytotoxicity Assay Kit was obtained from Beyotime (Shanghai, China). TUNEL Apoptosis Assay Kit was provided by Beyotime Institute of Biotechnology (Haimen, China). Ki67 antibody was provided by Cell Signaling Technology (MA, USA). 1 × Phosphate buffer solution (PBS) and deionized water were used in the experiments. All C57BL/6 female mice (18–20 g) were obtained from Yangzhou University Medical Center. All other reagents were purchased from Nanjing Wanqing Chemical Glassware Instrument Company and used as received.

### Synthesis of PCL-PEG-SS

We firstly synthesized HO-PCL-b-PEG^114^ with the terminal group of hydroxyl group. The specific synthesis steps are shown in Fig. 1. The synthesis of HO-PCL-b-PEG^114^ is carried out by using PEG^114^-OH as an initiator and stannous octoate (Sn(Oct)_2_) as a catalyst to initiate ring-opening polymerization of monomer ε-caprolactone (ε-CL). Specifically, 2 g of dry treated CH_3_O-PEG^114^-OH (0.4 mmol) and 4 g of de-vaporized ε-CL (35.1 mmol) were weighed and dissolved in 10 mL of vacuum-distilled anhydrous toluene, followed by one drop of Sn(Oct)_2_. The liquid nitrogen was frozen, evacuated, purged with nitrogen, thawed, and cycled three times. After reacting for 24 h in a 110 °C oil bath, it was added dropwise to 500 mL of ice diethyl ether under stirring to precipitate. It was suction filtered, washed with diethyl ether three times, and dried in vacuo to get white solid HO-PCL-b-PEG114. The degree of polymerization of the PCL segment was calculated by nuclear magnetic resonance to be Dp = 87. The block copolymer was HO-PCL87-b-PEG114. To synthesis PCL-PEG-SS, 0.5 g HO-PCL87-b-PEG114 were dissolved in 20 mL DMSO and thenadded 4-(dimethylamino) pyridine (DMPA, 0.2 g) solution in DMSO (3 mL) and lipoic acid anhydride (0.6 g) in DMSO (3 mL), respectively. The reaction was stirred for 48 h under nitrogen at 30 °C. The product was isolated by precipitation in cold ethanol, washed several times with ethanol, and dried in vacuo.

### Preparation of DOX&IR780@PEG-PCL-SS NPs

After hydrophobization of doxorubicin, certain amount of DOX (1.0 mg), IR780 (1.0 mg) and polymer PEG-PCL-SS (20 mg) were dissolved in DMSO, and then added to PBS (pH 7.4) under ultrasonic conditions. The solution was concentrated by ultrafiltration to remove free DOX and IR780. The drug loading was calculated by the drug amount in NPs, according to the standard curve of DOX and IR780 measured by UV absorption spectroscopy. Nanoparticles loaded with near-infrared photosensitizers were prepared by hydrophobic interaction of PCL and hydrophobic small molecules. The DTT was accurately weighed and the nanoparticles were reacted using DTT (10 μL, 10 mg/mL). Because DTT can partially hydrolyze the disulfide bond of the nanoparticles within a controllable range, the spatial structure of the nanoparticles after reflection is more compact. The nanoparticles loaded with photosensitizer and chemotherapeutic drugs were obtained by dialysis and concentration. As a control, non-crosslinked PEG-PCL NPs were prepared the same as PEG-PCL-SS NPs. To evaluate the delivery advantages of PEG-PCL-SS NPs, two other NPs (PLGA and Albumin) were prepared as described before [19–21]. Briefly, The PLGA NPs were prepared in a solvent displacement process. DOX (1 mg), IR780 (1 mg) and 20 mg PLGA first dissolved in DMSO (1 mL). 1 mL of the solution was added dropwise to 10 mL of water. The mixture was then stirred in open air for 2 h. Then the solution was concentrated by ultrafiltration to remove free DOX and IR780. DOX and IR780-loaded Albumin nanoparticles were prepared via a molecular switch method as described previously [21, 22]. The drug loading was calculated by the drug amount in NPs, according to the UV standard curve of DOX and IR780.

### Characterization of DOX&IR780@PEG-PCL-SS NPs

Particle size and surface potential were measured by a Brookhaven BI-90Plus laser particle size analyzer. Transmission electron microscopy was used to observe the morphology of the nanoparticles. Samples were prepared by dropping a suitable concentration of the nanoparticle solution on a 200 mesh copper mesh and dried overnight.

### Cell culture

Murine bladder carcinoma MB49 cells were obtained from Shanghai Institute of Biochemistry and Cell Biology, Chinese Academy of Sciences. The cell line was maintained in RPMI 1640 cell culture media supplemented with 10% fetal calf serum (Hyclone, Logan, UT) and antimicrobial-antimycotic (Gibco/Invitrogen, Carlsbad, CA) in a humidified incubator at 37 °C in an atmosphere composed of 5% CO_2_. The cell line was transduced with the firefly luciferase gene by a lentivirus vector.

### In vitro temperature curve

We prepared internal cross-linked polymeric nanoparticles containing IR780, internal cross-linked polymeric nanoparticles containing doxorubicin and IR780, and a PBS solution (pH7.4). The 808 nm near-infrared laser irradiation system was applied on the samples for 1–3 min, and the temperature probe measurement system was conducted. The photothermal effect of the IR780-containing nanoparticles was evaluated (50 μg/mL IR780).

### In vitro drug release

DOX and IR780 were encapsulated into the inner crosslinked polymeric nanoparticles. After dialysis, a small amount of organic solvent was removed. The dialysis bag filled with nanoparticles was placed in a PBS solution (pH7.4) with or without GSH (5 mM or 10 mM). The internal chemical structure of the internally crosslinked polymeric nanoparticles is changed when interacted with GSH, and the drugs are continuously released from the drug-loaded nanoparticles. We then used near-infrared laser irradiation to irradiate the cross-linked polymeric nanoparticles. The near-infrared laser can promote the photothermal reaction of IR780 in the drug-loaded nanoparticles, and further promote the release of the drug from the nanoparticles. The drug content of nanoparticles in PBS (pH 7.4) was measured at different time points (0 to 72 h) under different experimental conditions, and the drug release curve was obtained.

### Cellular uptake

MB49 cells were cultured for 24 h and then treated free doxorubicin solution (1.0 μg/mL), free IR780 solution (1.0 μg/mL), and drug-loaded nanoparticles containing doxorubicin and IR780for 6 h. The cells were washed with PBS twice and imaged by a fluorescence microscopy to determine the cellular uptake of the nanoparticles.

### In vitro cytotoxicity

Different concentrations of doxorubicin-containing nanoparticles (DOX, from 0.11 to 1.775 μg/mL), IR780-containing nanoparticles (IR780, from 0.078 to 1.25 μg/mL), drug-loaded nanoparticles containing doxorubicin and IR780, and nanoparticles without any drugs were added to the cells. After 24 h, the cell viability and optimum concentration of the formulations was evaluated by MTT assays by measuring the absorbance of the solution at 570 nm.

### In vitro therapeutic efficacy of DOX&IR780@PEG-PCL-SS NPs

Different concentrations of free doxorubicin solution, free IR780 solution, doxorubicin-containing nanoparticles, IR780-containing nanoparticles, doxorubicin and IR780 nanoparticles were prepared and added to the cells. The tumor cells were fully ingested with drugs or drug-loaded nanoparticles for 12 h, IR780 containing groups were irradiated with 808 nm near-infrared laser for 3 min, and then cultured for 24 h. MTT assays were conducted with the same procedure described previously.

### Establishment of an orthotopic bladder cancer model in C57BL/6 mice

All mice received care following the guidelines of the Care and Use of Laboratory Animals and their use followed the terms of the Institutional Animal Care regulations and Use Committee of Nanjing University. All animal experiments were approved by the Administration Committee of Experimental Animals in Jiangsu Province and the Ethic Committee of Nanjing University.

After anesthetizing C57BL/6 mice with 2% pentobarbital, the midline incision was taken to expose the bladder position of the mouse. After using the syringe to absorb the urine in the bladder, the MB49 bladder cancer cell suspension was injected into the bladder muscle layer. Small animal CT examination was performed at day 7 after successful surgery to observe whether there was a tumor in the bladder area.

### Pharmacokinetics study

Pharmacokinetics study following single-dose intravenous injection was conducted in tumor-free male mice. The mice were randomly divided into PEG-PCL-SS NPs group, PEG-PCL NPs group, PLGA NPs group and Albumin NPs group (three mice per group). The formulations were administered via intravenous injection with the IR780 dose of 10 mg/kg. Blood samples were collected by retro-orbital bleeding at different time points (1 to 72 h) after administration. The content of IR780 in the serum samples was measured using a Varioskan Flash Spectral Scanning multimode plate reader (Thermo Fisher Scientific, Waltham, MA, USA). PK Solver Version 2.0, was used to calculate pharmacokinetic parameters from the plasma concentration versus time data [23].

### In vivo biodistribution

The free IR780 solution and the nanoparticles loaded with doxorubicin and IR780 (0.3 mg IR780/kg body weight) were injected into the tumor-bearing mice by tail vein administration. Images were taken at 6, 12, 24 and 48 h after injection using the in vivo imaging system (IVIS Lumina XR III, USA). As a control, normal mice (without tumor) were injected with DOX&IR780@PEG-PCL-SS NPs and imaged at 6, 12, 24 and 48 h after injection using the in vivo imaging system. The mice were sacrificed at 48 h after injection, and the major organs including the heart, liver, spleen, lung, kidney and bladder tumor were collected for ex vivo imaging. The excitation wavelength of IR-780 is 745 nm and its emission spectrum is 780–900 nm.

### Therapeutic efficacy of DOX&IR780@PEG-PCL-SS NPs using orthotopic bladder cancer model

The luciferase-expressing MB49 cell line was constructed and used for in vivo imaging of the tumor. Saline, doxorubicin-containing nanoparticles (4 mg/kg IR780), IR780-containing nanoparticles (2.5 mg/kg DOX), "drug-loaded nanoparticles containing doxorubicin and IR780" (2.5 mg/kg DOX, 4 mg/kg IR780) were injected into randomized mouse groups by tail vein administration. The experimental groups were irradiated with 808 nm near-infrared laser after 24 h, and repeated administration after one week. The mice were sacrificed after 3 weeks, and the heart, liver, spleen, lung, kidney and bladder of the mice were harvested for further study.

### In vivo toxicity study

Polymeric nanoparticles loaded with different concentrations of doxorubicin (0 mg/kg, 1.25 mg/kg, 2.5 mg/kg and 5.0 mg/kg) and IR780 (0 mg/kg, 1.25 mg/kg, 2.5 mg/kg and 5.0 mg/kg) were synthesized and injected into the mice by tail vein administration. After 24 h, the mice were sacrificed and blood samples were harvested by retro-orbital bleeding. ALT, AST, BUN, and Cr in the blood samples were immediately detected.

### Histology

After the mice were sacrificed, the heart, liver, spleen, lung, kidney and bladder of the mice were harvested for hematoxylin and eosin (H&E) staining, the TUNEL assay and Ki67 immunofluorescence. After dehydration and fixation, the sections were stained with H&E, and the bladder and tumor tissues were stained with TUNEL to observe the apoptosis in the bladder cancer tissues. Briefly for the TUNEL assay, the sections were fully deparaffinized and hydrated. 20 μg/mL DNase-free proteinase K was added to the samples and kept them at room temperature for 30 min. After washing twice in PBS, samples were incubated with 50 μL of TUNEL inspection fluid for 60 min before rinsed three times with PBS. Results were imaged under a fluorescent microscopy by using 488 nm excitation and 530 nm emission. For Ki67 immunofluorescence study, the sections were fully deparaffinized and hydrated, and the tissue was permeabilized with 0.3% triton X-100 (prepared in PBS) for 10 min at room temperature, and blocked with 5% BSA for 1 h. Use the corresponding antibody diluent to dilute the primary antibody and incubate at room temperature for 3 h. Wash the sections with PBS, use the corresponding antibody diluent to dilute the secondary antibody, incubate at room temperature for 60 min, and observe after mounting. Results were imaged under a fluorescent microscopy by using 585 nm excitation and 624 nm emission.

## Results and discussion

### Preparation and characterization of DOX&IR780@PEG-PCL-SS NPs

The chemical synthesis process of PEG-PCL-SS conjugates was illustrated in Fig. 1. Firstly, The synthesis of HO-PCL-b-PEG114 is carried out by using PEG114-OH as an initiator and stannous octoate (Sn(Oct)_2_) as a catalyst to initiate ring-opening polymerization of monomer ε-caprolactone (ε-CL). Then, to synthesis PCL-PEG-SS, HO-PCL-b-PEG114 reacted with DMPA and lipoic acid anhydride for 48 h (Fig. 1a). Drug loaded nanoparticles were prepared through a co-precipitation method. Briefly, hydrophobizated doxorubicin, IR780 and polymer PCL-PEG-SS dissolved in DMSO, were added to PBS (pH7.4) under ultrasonic conditions. Nanoparticles loading with near-infrared photosensitizers and chemotherapeutics were formed by hydrophobic interaction of PCL part of PCL-PEG-SS and hydrophobic small molecules. Under DTT catalysis condition, the intramolecular disulfide bonds of PCL-PEG-SS will be broken, while intermolecular disulfide bonds may form inside the nanoparticles through the disulphide–sulfhydryl interchange reaction and stabilize the nanoparticles, which makes the spatial structure of nanoparticles more compact (Fig. 1b). The resulting cross-linked PCL-PEG-SS nanoparticles are stable under high salt concentration.

**Fig. 2:**
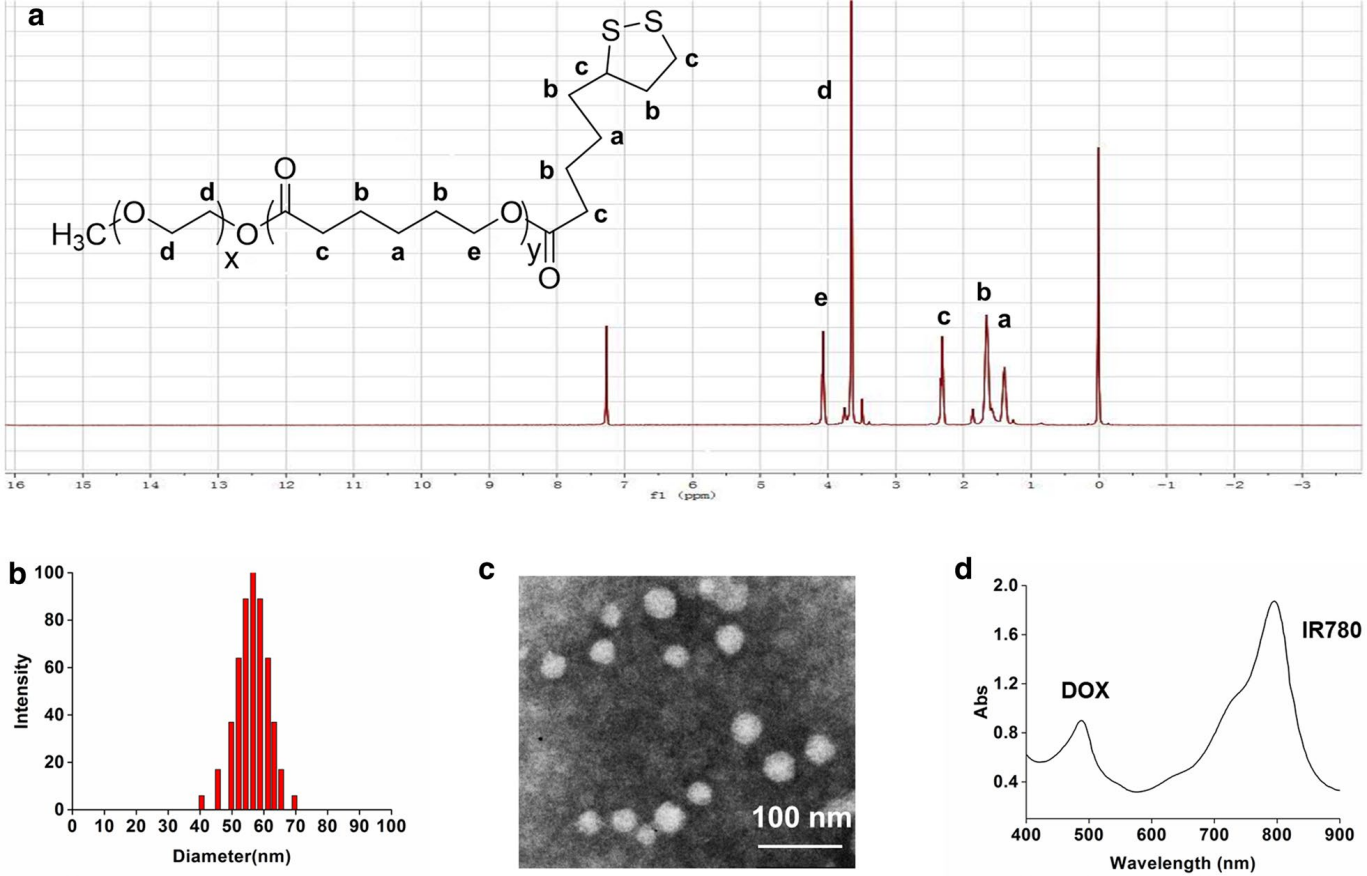
Characterization of cross-linked PEG-PCL-SS nanoparticles. **a** Chemical structure and ^1^H-NMR spectra of PEG-PCL-SS copolymer. **b** Size distribution of cross-linked PEG-PCL-SS nanoparticles. **c** Transmission electron microscopy (TEM) image of PEG-PCL-SS nanoparticles. **d** Optical properties of IR780 and DOX-loaded PEG-PCL-SS nanoparticles

The characteristics of the DOX&IR780@PEG-PCL-SS NPs are shown in Fig. 2. The results of ^1^H NMR result prove that we synthesized the carrier PEG-PCL-SS (Fig. 2a). The drug loading of IR780 and DOX in NPs were 4.3% and 3.8% respectively. The average size (tested by DLS) of DOX&IR780@PEG-PCL-SS NPs was 59.24 ± 18.47 nm and DOX&IR780@PEG-PCL-SS NPs were generally spherical in shape as shown in the TEM image (Fig. 2b, c). To compare the size change of PEG-PCL-SS NPs, cross-linked PEG-PCL-SS NPs and drug-loaded PEG-PCL-SS NPs were prepared. The particle size change of all these NPs was shown in Additional file 1: Figure S1. When DTT was added to PEG-PCL-SS NPs or DOX&IR780@PEG-PCL-SS NPs, the particle sizes were reduced because of internal cross-linking ability of disulfide bond. Full wavelength absorption of DOX&IR780@PEG-PCL-SS NPs shows that the nanoparticles have two absorption peaks at 497 nm and 780 nm, which are the characteristics of doxorubicin and IR780, respectively. While DOX and IR780 are hydrophobic, the UV spectrum proves that both drugs successfully assembled into nanoparticles. XPS experiment was carried out here to evaluate the superficial elements of DOX&IR780@PEG-PCL-SS NPs. From the results shown in Additional file 1: Figure S2, a significant Cl peak was observed in free DOX, while “I” element peak was observed in IR780. When IR780 and DOX were encapsulated into PEG-PCL-SS NPs, the intensity of O and N peak did not change greatly. However, the intensity of Cl and “I” peaks were significantly lower than that of free DOX, free IR780, or physical mixture. These results indicated that the superficial elements of DOX&IR780@PEG-PCL-SS NPs were mainly composed of PEG-PCL-SS while the DOX and IR780 were mostly located inside nanoparticles.

### In vitro temperature curve and stability study

**Fig. 3:**
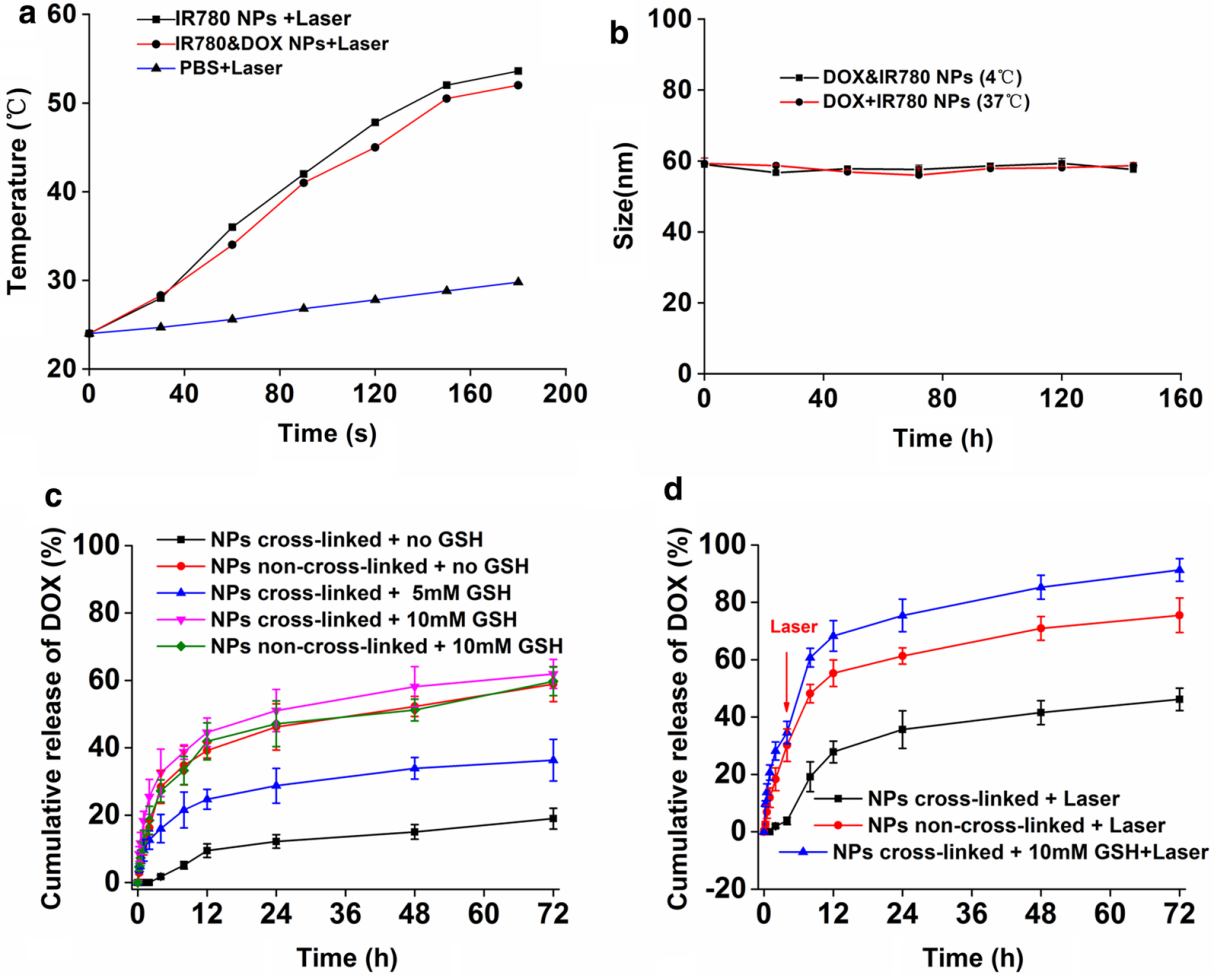
**a** The temperature curve of PBS, IR780 nanoparticles and IR780&DOX nanoparticles under NIR laser irradiation (808 nm, 1.6 W/cm^2^). **b** Stability of IR780&DOX nanoparticles at 4℃ and 37℃. **c** The cumulative release of DOX from micelles in different concentrations of GSH solution. **d** The effects of laser irradiation (808 nm, 1.6 W/cm^2^) on the DOX release rate (n = 3)

IR780 can be used as an effective photothermal agent. Under 808 nm laser irradiation, the IR780 NPs and DOX&IR780 NPs (50 μg/mL IR780) can increase the temperature over time, while the temperature of PBS group was only slightly increased. It demonstrated that IR780 has a significant photothermal effect (up to 53.6 °C) and the addition of DOX does not affect the photothermal effect (Fig. 3a). The prepared nanoparticles were then placed into the environment at 37 °C and 4 °C and particle sizes were measured every 24 h (Fig. 3b). The results show that the nanoparticles have excellent stability for at least 144 h at 4 °C or body temperature. To prove the advantage of PEG-PCL-SS NPs, two other common carriers-based (PLGA and Albumin) nanoparticles were prepared [24]. After PEGylation, the size of PEG-PCL-SS NPs was about 60 nm, smaller than PLGA and Albumin NPs (Additional file 1: Figure S3). For the stability study, both PEG-PCL, PLGA and albumin NPs will precipitate in one week, while PEG-PCL-SS NPs can keep stable in one week.

### In vitro drug release

An in vitro drug release study was conducted to confirm the duel responsiveness of the nanoparticles to GSH and photothermal effect. GSH was added to the PBS to mimic the tumor cell microenvironment. It is important to note that as glutathione (5 mM to 10 mM) increases, DOX in the nanoparticles can be released more and more quickly (from 31.3% to 61.9%) (Fig. 3c). This is because the breakdown of the disulfide bond in the nanoparticle structure due to GSH can result in the drug release. In addition, we have found that with the photothermal effect produced by the addition of the photosensitizer IR780, the release of the drug can be greatly promoted (Fig. 3d). This experiment validated the properties of nanoparticles with controlled release manner, and we can trigger drug release with glutathione and near-infrared laser irradiation. According to previous studies, the invasive bladder cancer normally contains higher amount of glutathione. Therefore, the nanoparticles we developed can provide controlled drug release in a GSH-rich environment and potentially enhance therapeutic effects against tumors with high malignancy.

### In vitro cytotoxicity and therapeutic efficacy

**Fig. 4:**
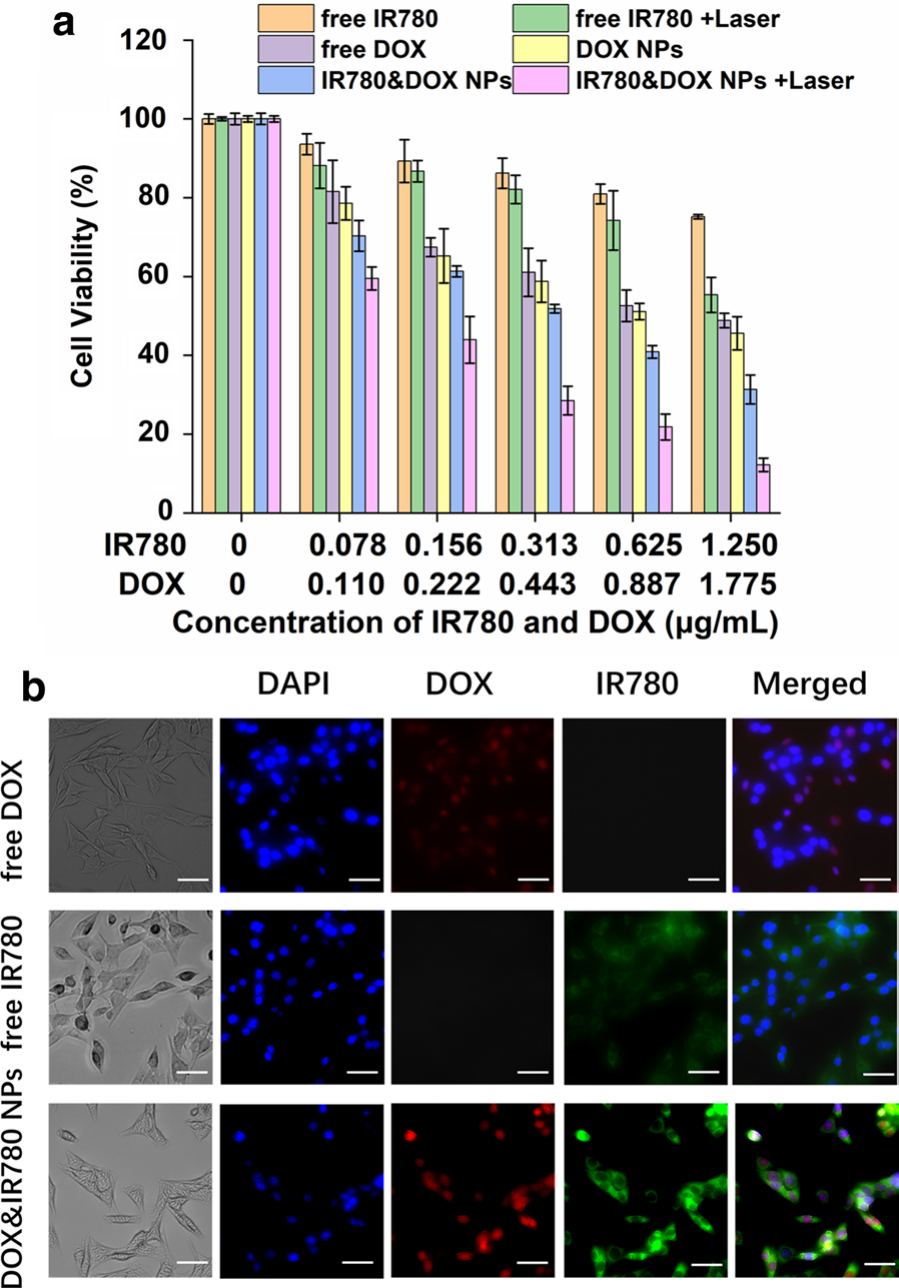
Cell viability and uptake of IR780&DOX nanoparticles. **a** In vitro cytotoxicity and therapeutic efficacy of the IR780 and DOX-loaded PEG-PCL-SS nanoparticles. **b** Observing cellular uptake using a fluorescence microscope in bright field, DOX channel, IR780 channel, DAPI and merge vision. Scale bars = 20 μm. (eyepiece: 1×, objective lens: 20×)

The cell cytotoxicity of the drug-loaded nanoparticles was evaluated by the MTT assay (Fig. 4a). Under the near-infrared laser irradiation, the cytotoxicity produced by the photothermal effect was significantly higher than that of the other control groups (from IR780 concentration of 0.078 μg/mL or more). The results confirmed that the nanoparticles had a good photothermal treatment effect. Both free IR780 and DOX exhibited antitumor ability in cancer cells. However, the NPs co-loaded with IR780 and DOX showed strong, synergistic therapy for cancer treatment. In the meantime, the cytotoxicity of NPs in normal cells is much less than that of cancer cells (Fig. 4a, Additional file 1: Figure S4). However, as DOX is very toxic, and the photothermal effect of IR780 is very strong, the cytotoxicity of NPs in normal cells is clearly observed. The difference of toxicity is mainly caused by the difference of cell uptake. The drug amount of cancer cells uptake is much higher than normal cells. When IR780 concentration was 1.25 μg/mL and DOX was 1.775 μg/mL, cell viability was 31.34% and 12.22% without or with laser irradiation, respectively. At the same time, it can be observed that the difference in cytotoxicity during the process of increasing DOX concentration is getting smaller and smaller, indicating that the effect of chemotherapeutic drugs begins to play a leading role as the concentration of doxorubicin increases. This is consistent with the clinical situation.

### Cellular uptake

To monitor cellular uptake of DOX&IR780@PEG-PCL-SS NPs, the nanoparticles were incubated with MB-49 cancer cells, and the result was observed by fluorescence microscope. As can be seen from Fig. 4b, compared with free DOX or free IR780 group, DOX&IR780@PEG-PCL-SS NPs had more DOX and IR780 fluorescence within the cells, indicating NPs enhanced the drug uptake. This is beneficial for the photothermal and chemotherapy.

### In vivo distribution and pharmacokinetics of nanoparticles

**Fig. 5:**
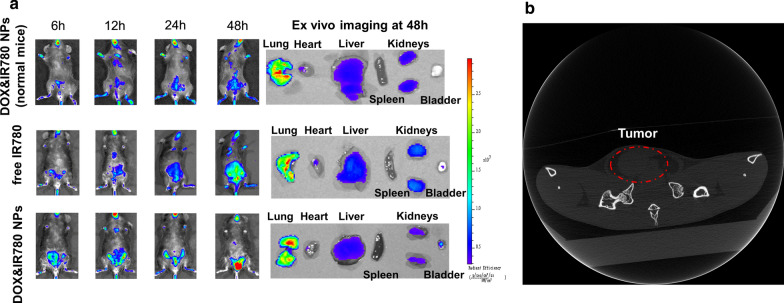
Establishment of an orthotopic bladder cancer model in C57BL/6 mice and in vivo distribution of free IR780 and DOX&IR780@PEG-PCL-SS NPs. **a** The upper is the biodistribution of NPs in normal mouse. The below is the In vivo distribution of free IR780 and DOX&IR780@PEG-PCL-SS NPs in cancer mice. The right size is ex vivo imaging of NPs in lung, heart, liver, spleen, kidney and bladder of the mice. **b** Through small animal CT, a tumor is shown when the bladder of the mouse is filled with urine 7 days after MB49-luciferase cells inject into the bladder wall

In order to verify the accumulation of nanoparticles in tumor, we investigated the biodistribution of the nanoparticle in a mouse bladder cancer model. As can be seen from Fig. 5a, free IR780 also has a certain accumulation in bladder tumors, which is the passive accumulation of the near-infrared dye itself. For the normal mouse group, there is part of NPs accumulating in the bladder region. Ex vivo imaging (the last organ is bladder for each group) showed that bladder had very few NPs compared with other organs. The nanoparticle group accumulates in the tumor, indicating that our nanoparticles have good tumor targeting. The distribution of DOX&IR780@PEG-PCL-SS NPs provides a solid foundation for further treatment. PEGylation is a common approach for improving the efficiency of drug delivery in vivo. In vivo pharmacokinetics of different nanoparticles were compared here. From the results (Additional file 1: Figure S5), there are no significant difference in half-life time (t_1/2_) of PEG-PCL-SS, PEG-PCL and albumin NPs (24.18 h vs 20.63 h vs 22.37 h). The half-life time of PLGA NPs is 6.07 h, much less than other three carriers. PEGylation improved the in vivo circulation time of drug loaded NPs. Albumin is a classic drug carrier, and paclitaxel loaded albumin NPs are used in clinic [23]. The results showed that PEG-PCL-SS as a drug carrier is comparable with Albumin.

### Therapeutic efficacy of DOX&IR780@PEG-PCL-SS NPs in orthotopic bladder cancer model

We established the orthotopic bladder cancer model in C57BL/6 mice according to our previously reported method [2]. Through small animal CT, a tumor can be observed when the bladder of the mouse is filled with urine (Fig. 5b). This indicated the successful construction of a mouse orthotopic bladder cancer model.

**Fig. 6:**
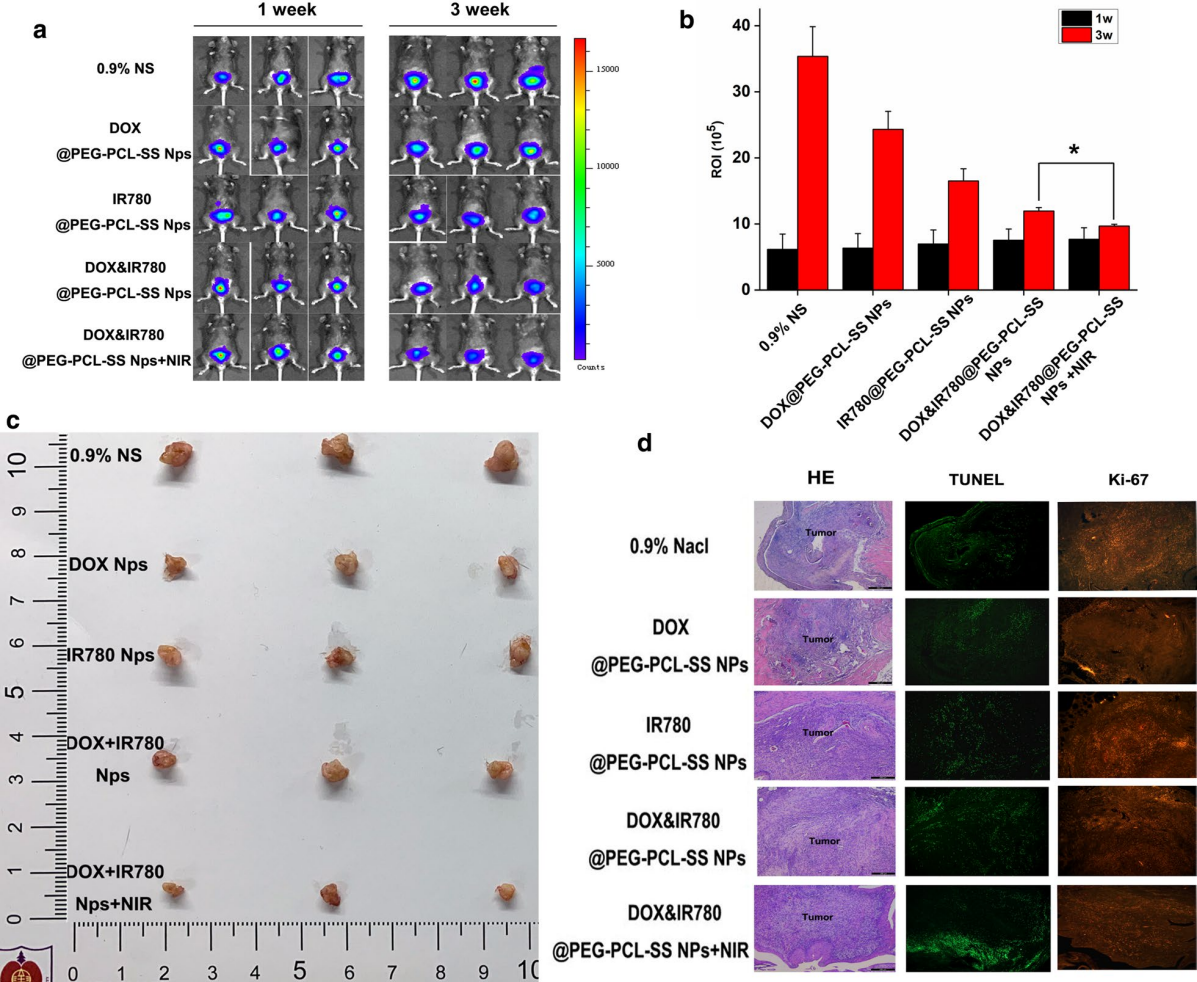
Nanoparticles antitumor activity research. **a** bioluminescence of mouse tumor cells in 1 week and 3 weeks after model administration. **b** Statistical graph of ROI values for bioluminescence imaging. *P < 0.05, vs indicated groups. **c** Size map of tumors taken at the third week after model administration. **d** HE, TUNEL and Ki-67 staining of mouse bladder tumor tissue (eyepiece: 1×, objective lens: 5×)

Changes in tumor size in a mouse bladder cancer model can be observed by In vivo imaging. One week after injection of luciferase-expressing MB49 cells, significant bioluminescence aggregation in the bladder area of mice was observed, suggesting tumor formed (Fig. 6a). After 3 weeks’ treatment, mice treated with DOX&IR780@PEG-PCL-SS NPs and near-infrared laser irradiation achieved the best anticancer effect, and the tumor growth was the slowest compared with other groups, indicating that the combined therapy can achieve synergistic inhibition effect for the bladder tumors. We then used the ROI value of fluorescence imaging to further evaluate the size of bladder tumors (Fig. 6b), and quantitatively confirmed the reduction of bladder tumor sizes after the treatment using DOX&IR780@PEG-PCL-SS NPs and near-infrared laser irradiation. Additionally, visual photos of the bladder tumors of the mice obtained in the third week (21 days) were taken by camera and the results are consistent with in vivo imaging data (Fig. 6c). Photographs of normal bladder and orthotopic bladder cancer are shown in Fig. 6c. The normal bladder wall was transparent with no solid lesions. An obvious solid lesion was observed in the bladder cancer model, showing the carcinoma invasion in all layers of the bladder wall, including the mucosa and muscular layer. This orthotopic bladder cancer model was similar to muscular invasive bladder cancer of humans in the clinic.

### Organ sectioning and staining

After the mice were sacrificed, the heart, liver, spleen, lung, kidney and bladder of the mice were collected for histological analysis. The bladder and tumor tissues were stained with H&E, TUNEL or Ki-67 to observe the apoptosis and cell proliferation in the bladder cancer tissues. The results of bladder tumor staining showed that the bladder tumor involved the whole bladder layer, and the tumor apoptosis level of the tumors from the mice treated with DOX&IR780@PEG-PCL-SS NPs combined with near-infrared laser irradiation was significantly higher than that of other experimental groups while the bladder cancer cell proliferation was less than other groups (Ki67 staining results) (Fig. 6d).

### In vivo toxicity and biosafety

**Fig. 7:**
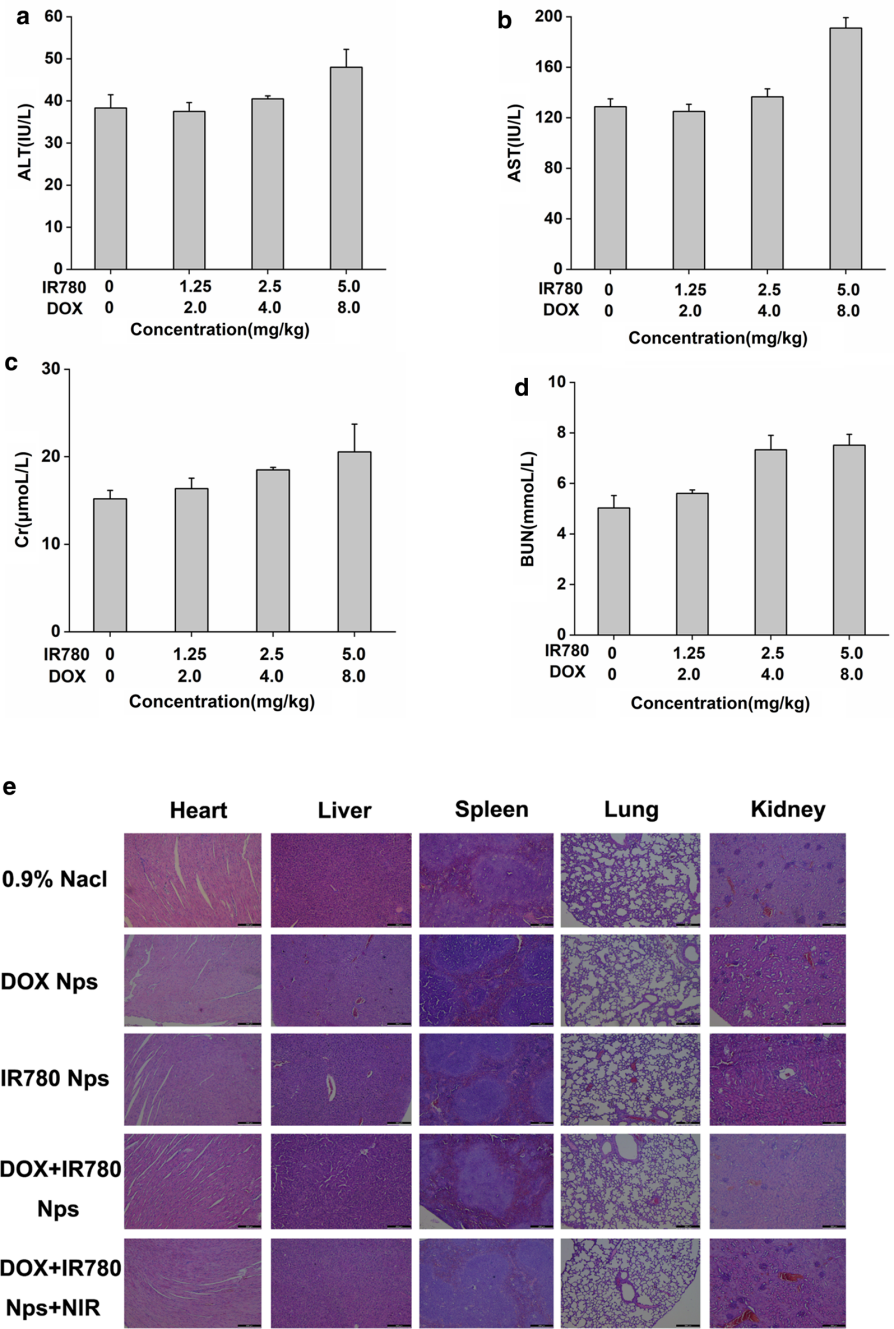
In vivo cytotoxicity and organ safety evaluation**.** 24 h after administration, the mice liver and kidney function tests: **a** ALT test, **b** AST test, **c** Cr test, **d** BUN test. **e** Organ safety evaluation in mouse heart, liver, spleen, lung and kidney. Scale bar = 100 μm. (eyepiece: 1×, objective lens: 5×)

To evaluate the toxicity of the nanoparticles at the whole-body level, blood samples and major organs were harvested for serological and histological analysis. There were no obvious accumulation of nanoparticles and functional damage. 24 h after the tail vein administration of the mice, blood was obtained by eyeball blood sampling. Serological tests in mice showed that the selected nanoparticle concentration (from 0 to 2.5 mg/kg DOX, from 0 to 4 mg/kg IR780) did not identify liver and kidney damage. However, significant changes in liver and kidney function were observed after injection of high concentrations of nanoparticles (5 mg/kg DOX, 8 mg/kg IR780) (Fig. 7a–d). The major organs (heart, liver, spleen, lung, and kidney) of all the groups showed no noticeable histological changes, suggesting the nanoparticles have no toxicity to the organs and are biocompatible to be used as drug formulations (Fig. 7e).

## Conclusions

In this study, we successfully fabricated the DOX&IR780@PEG-PCL-SS NPs for delivering IR780 and DOX, which could achieve NIR laser-controlled drug release and imaging guidance for chemo-photothermal synergistic therapy for bladder cancer. The average size of the polymeric nanoparticles was 59.24 ± 18.47 nm and stable for at least 144 h at body temperature. The DOX&IR780@PEG-PCL-SS NPs have dual sensitivity to high levels of glutathione in bladder cancer cells and near-infrared laser irradiation, leading to the ability of nanoparticles to have good photothermal properties and bladder tumor tissue targeting. Under the action of near-infrared laser irradiation in glutathione-rich environment, nanoparticles can control the release of drugs and effectively destroy tumor cells. After 21 days, the DOX&IR780@PEG-PCL-SS NPs with near-infrared laser irradiation experimental group can significantly reduce tumor size and inhibit their growth compared to the control group based on the in vivo therapeutic efficacy study. It is obvious that nanoparticles have good inhibition effects against orthotopic bladder tumors. We propose that our nanoparticles can be a promising treatment strategy for bladder cancer (especially for the locally aggressive lesions) photothermal ablation with an enormous potential for clinical translation.

## Supplementary information

**Additional file 1: Figure S1.** Size change of PEG-PCL-SS NPs, cross-linked PEG-PCL-SS NPs and drug-loaded PEG-PCL-SS NPs. **Figure S2.** XPS spectra of five samples (free DOX, free IR780, physical mixture, PEG-PCL-SS and DOX&IR780@PEG-PCL-SS NPs). **Figure S3.** Particle size and stability of different nanoparticles. PEG-PCL NPs and PLGA NPs precipitated after 72 h while Albumin NPs precipitated after one week. **Figure S4.** Cell viability of IR780&DOX nanoparticles in bladder normal mucosa cells (SV-HUC-1). **Figure S5.**  Pharmacokinetics of different carriers-based NPs in mice after intravenous injection determined based on IR780 absorption. A–C) Blood circulation curves of PEG-PCL-SS, PEG-PCL, Albumin and PLGA NPs at interval times (*n* = 3). PK Solver Version 2.0, was used to calculate pharmacokinetic parameters.

## Data Availability

The datasets used and analyzed during the current study are available from the corresponding author on reasonable request.

## Abbreviations

DOX: doxorubicin
DTT: dithiothreitol
HE: hematoxylin–eosin staining
TUNEL: terminal deoxynucleotidyl transferase-mediated dUTP-biotin nick end labeling assay
ALT: alanine aminotransferase
AST: aspartate aminotransferase
Cr: creatinine
BUN: blood urea nitrogen

## References

[1] Burger M, Catto JW, Dalbagni G, Grossman HB, Herr H, Karakiewicz P (2013). Epidemiology and risk factors of urothelial bladder cancer. Eur Urol.

[2] Lin T, Zhao X, Zhao S, Yu H, Cao W, Chen W (2018). O2-generating MnO2 nanoparticles for enhanced photodynamic therapy of bladder cancer by ameliorating hypoxia. Theranostics.

[3] Witjes JA, Comperat E, Cowan NC, De Santis M, Gakis G, Lebret T (2014). EAU guidelines on muscle-invasive and metastatic bladder cancer: summary of the 2013 guidelines. Eur Urol.

[4] Kurth KH, Bouffioux C, Sylvester R, van der Meijden AP, Oosterlinck W, Brausi M (2000). Treatment of superficial bladder tumors: achievements and needs.. The EORTC Genitourinary Group. Eur Urol.

[5] Antoni S, Ferlay J, Soerjomataram I, Znaor A, Jemal A, Bray F (2017). Bladder cancer incidence and mortality: a global overview and recent trends. Eur Urol.

[6] Parekh DJ, Bochner BH, Dalbagni G (2006). Superficial and muscle-invasive bladder cancer: principles of management for outcomes assessments. J Clin Oncol.

[7] Melamed JR, Edelstein RS, Day ES (2015). Elucidating the fundamental mechanisms of cell death triggered by photothermal therapy. ACS Nano.

[8] Hahn GM, Braun J, Har-Kedar I (1975). Thermochemotherapy: synergism between hyperthermia (42–43 degrees) and adriamycin (of bleomycin) in mammalian cell inactivation. Proc Natl Acad Sci USA.

[9] Li Z, Wang H, Chen Y, Wang Y, Li H, Han H (2016). pH- and NIR light-responsive polymeric prodrug micelles for hyperthermia-assisted site-specific chemotherapy to reverse drug resistance in cancer treatment. Small.

[10] Zheng M, Yue C, Ma Y, Gong P, Zhao P, Zheng C (2013). Single-step assembly of DOX/ICG loaded lipid–polymer nanoparticles for highly effective chemo-photothermal combination therapy. ACS Nano.

[11] Zhang P, Hu C, Ran W, Meng J, Yin Q, Li Y (2016). Recent progress in light-triggered nanotheranostics for cancer treatment. Theranostics.

[12] Lin T, Yuan A, Zhao X, Lian H, Zhuang J, Chen W (2017). Self-assembled tumor-targeting hyaluronic acid nanoparticles for photothermal ablation in orthotopic bladder cancer. Acta Biomater.

[13] Lafuente A, Giralt M, Cervello I, Pujol F, Mallol J (1990). Glutathione-S-transferase activity in human superficial transitional cell carcinoma of the bladder. Comparison with healthy controls. Cancer.

[14] Giralt M, Lafuente A, Pujol F, Mallol J (1993). Enhanced glutathione s-transferase activity and glutathione content in human bladder cancer. Followup study: influence of smoking. J Urol.

[15] Loras A, Suárez-Cabrera C, Martínez-Bisbal MC, Quintás G, Paramio JM, Martínez-Máñez R (2019). Integrative metabolomic and transcriptomic analysis for the study of bladder cancer. Cancers.

[16] Zhang L, Liu F, Li G, Zhou Y, Yang Y (2015). Twin-arginine translocation peptide conjugated epirubicin-loaded nanoparticles for enhanced tumor penetrating and targeting. J Pharm Sci.

[17] Zhao Y, Zhao W, Lim YC, Liu T (2019). Salinomycin-Loaded gold nanoparticles for treating cancer stem cells by ferroptosis-induced cell death. Mol Pharm.

[18] Yue C, Liu P, Zheng M, Zhao P, Wang Y, Ma Y (2013). IR-780 dye loaded tumor targeting theranostic nanoparticles for NIR imaging and photothermal therapy. Biomaterials.

[19] Gong G, Zhi F, Wang K, Tang X, Yuan A, Zhao L (2011). Fabrication of a nanocarrier system through self-assembly of plasma protein and its tumor targeting. Nanotechnology.

[20] Hu CM, Zhang L, Aryal S, Cheung C, Fang RH, Zhang L (2011). Erythrocyte membrane-camouflaged polymeric nanoparticles as a biomimetic delivery platform. Proc Natl Acad Sci USA.

[21] Wang K, Yuan A, Yu J, Wu J, Hu Y (2016). One-step self-assembling method to prepare dual-functional transferrin nanoparticles for antitumor drug delivery. J Pharm Sci.

[22] Zhao Y, Chen G, Meng Z, Gong G, Zhao W, Wang K (2019). A novel nanoparticle drug delivery system based on PEGylated hemoglobin for cancer therapy. Drug Deliv.

[23] Wang K, Zhang Y, Wang J, Yuan A, Sun M, Wu J (2016). Self-assembled IR780-loaded transferrin nanoparticles as an imaging, targeting and PDT/PTT agent for cancer therapy. Sci Rep.

[24] Zhu H, Zhang S, Ling Y, Meng G, Yang Y, Zhang W (2015). pH-responsive hybrid quantum dots for targeting hypoxic tumor siRNA delivery. J Control Release.

