# Supplementary material for: Internal cross-linked polymeric nanoparticles with dual sensitivity for combination therapy of muscle-invasive bladder cancer
Source: J Nanobiotechnology. 2020 Sep 4;18:124. doi: 10.1186/s12951-020-00686-3 (PMC7472706; doi:10.1186/s12951-020-00686-3)
Supplement: Supplementary file 1 — Additional file 1: Figure S1. Size change of PEG-PCL-SS NPs, cross-linked PEG-PCL-SS NPs and drug-loaded PEG-PCL-SS NPs. Figure S2. XPS spectra of five samples (free DOX, free IR780, physical mixture, PEG-PCL-SS and DOX&IR780@PEG-PCL-SS NPs). Figure S3. Particle size and stability of different nanoparticles. PEG-PCL NPs and PLGA NPs precipitated after 72 h while Albumin NPs precipitated after one week. Figure S4. Cell viability of IR780&DOX nanoparticles in bladder normal mucosa cells (SV-HUC-1). Figure S5. Pharmacokinetics of different carriers-based NPs in mice after intravenous injection determined based on IR780 absorption. A–C) Blood circulation curves of PEG-PCL-SS, PEG-PCL, Albumin and PLGA NPs at interval times (n = 3). PK Solver Version 2.0, was used to calculate pharmacokinetic parameters. [file 12951_2020_686_MOESM1_ESM.docx]

**Internal cross-linked polymeric nanoparticles with dual sensitivity for combination therapy of muscle-invasive bladder cancer**

Guanchen Zhu^1#^, KaikaiWang^2#^, Haixiang Qin^1^, Xiaozhi Zhao^1^, Wei Chen^1^, Linfeng Xu^1^, Wenmin Cao^1^, Hongqian Guo^1*^

^1^Department of Urology, Affiliated Drum Tower Hospital, Medical School of Nanjing University, Nanjing 210009, China.

^2^School of Pharmacy, Nantong University, Nantong 226001, China

^#^ These authors contribute equally

^*^Corresponding author: dr.ghq@nju.edu.cn (Hongqian Guo)


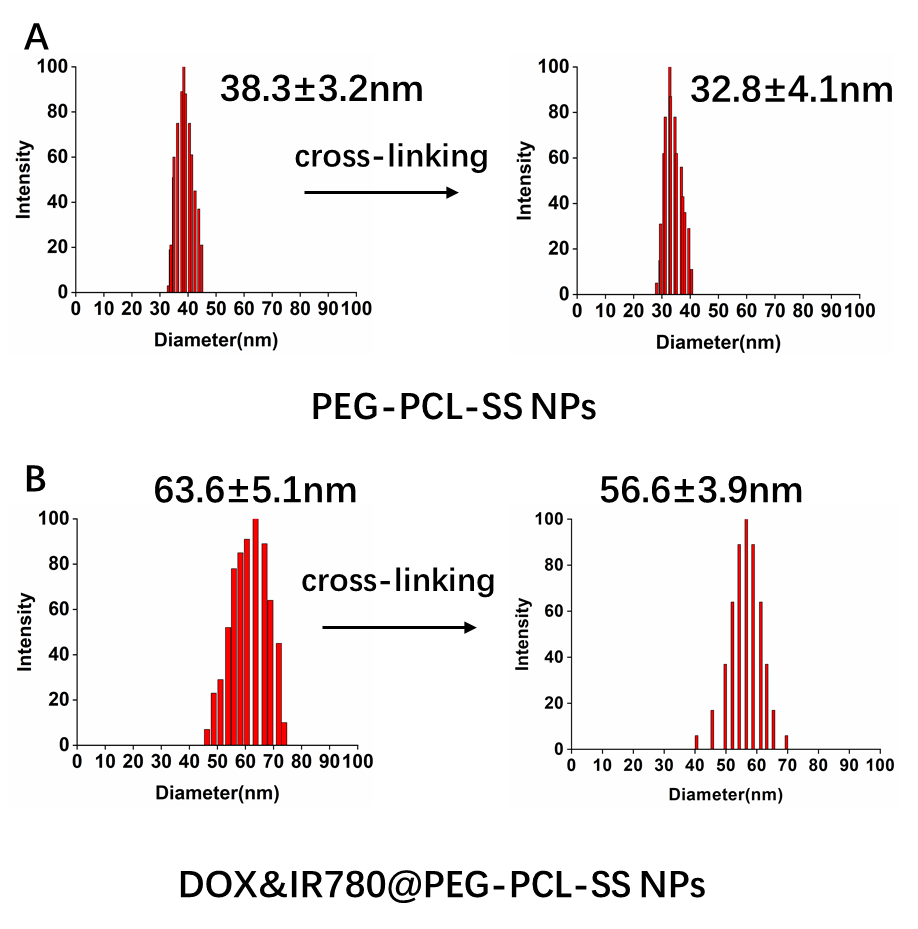


Figure S1. Size change of PEG-PCL-SS NPs, cross-linked PEG-PCL-SS NPs and drug-loaded PEG-PCL-SS NPs.


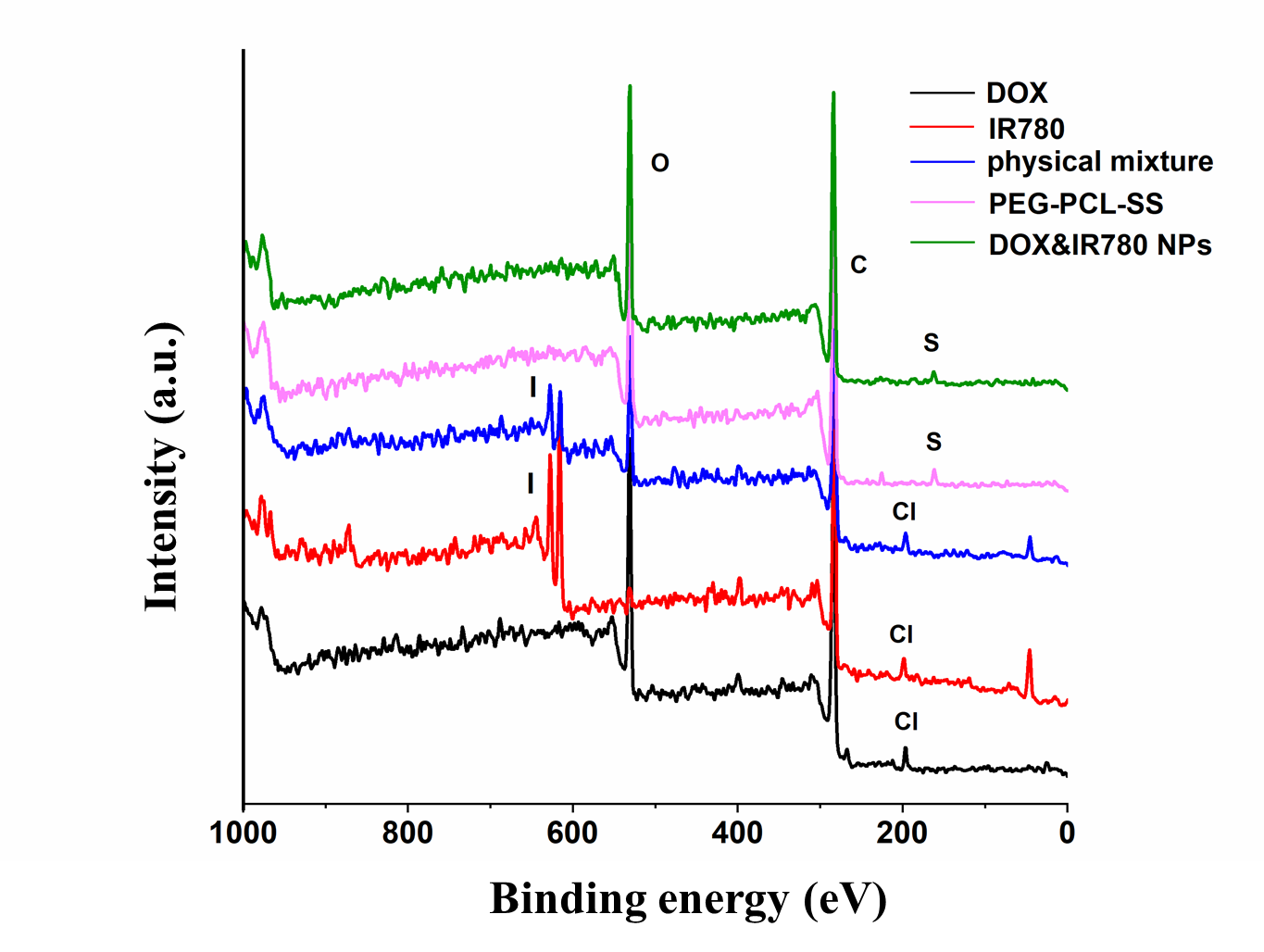


Figure S2. XPS spectra of five samples (free DOX, free IR780, physical mixture, PEG-PCL-SS and DOX&IR780@PEG-PCL-SS NPs).


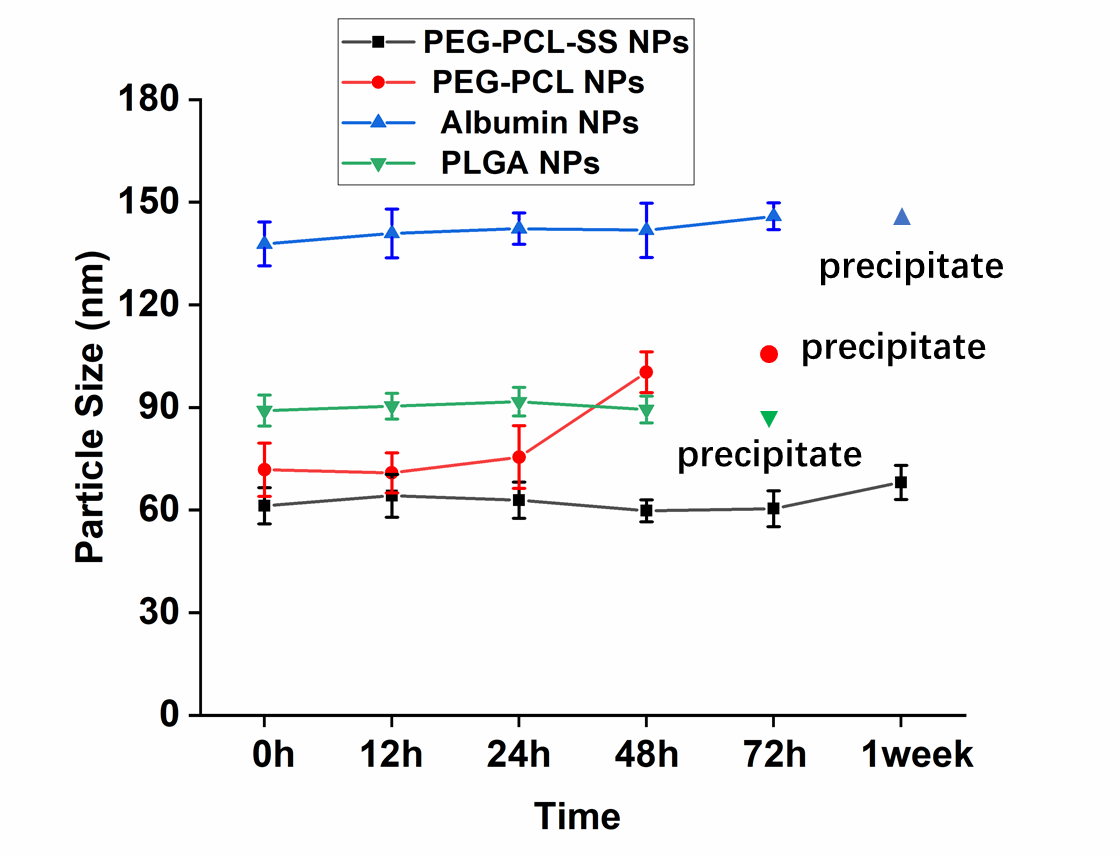


Figure S3. Particle size and stability of different nanoparticles. PEG-PCL NPs and PLGA NPs precipitated after 72 hours while Albumin NPs precipitated after one week.





Figure S4. Cell viability of IR780&DOX nanoparticles in bladder normal mucosa cells (SV-HUC-1).


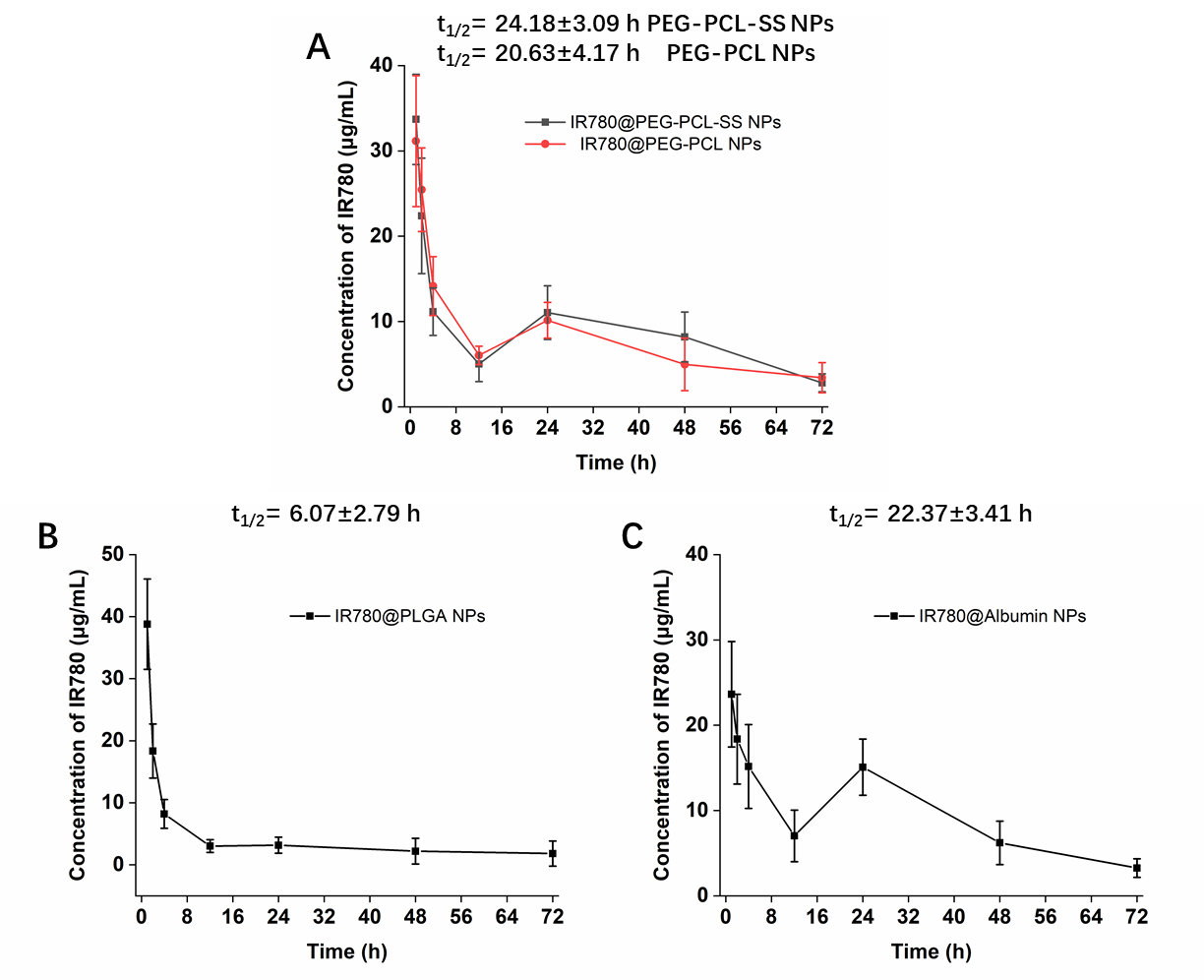


Figure S5. Pharmacokinetics of different carriers-based NPs in mice after intravenous injection determined based on IR780 absorption. A-C) Blood circulation curves of PEG-PCL-SS, PEG-PCL, Albumin and PLGA NPs at interval times (n=3). PK Solver Version 2.0, was used to calculate pharmacokinetic parameters [1].

Reference

[1] Zhang, Y., Huo, M., Zhou, J. & Xie, S. PKSolver: An add-in program for pharmacokinetic and pharmacodynamic data analysis in Microsoft Excel. Comput Methods Programs Biomed 99, 306-314 (2010).
